# Cost-effective treatment for diabetic macular edema using dexamethasone sodium phosphate

**DOI:** 10.5935/0004-2749.2024-0350

**Published:** 2024-12-18

**Authors:** Rodrigo Pessoa Cavalcanti Lira, Armando Ykaro Soares de Oliveira, Gabriel Rocha Lira

**Affiliations:** 1 Universidade Federal de Pernambuco, Recife, PE, Brazil

Dear Editor,

Diabetic macular edema (DME) is the leading cause of visual loss secondary to diabetic retinopathy^(^1^,^2^)^. In patients with DME, a long-acting dexamethasone release system (0.70 mg; Ozurdex) reduces the central macular thickness (CMT) over 3-4 months. However, it is expensive and can significantly increase the intraocular pressure (IOP) in up to 30% of the patients^(^3^)^. The price of dexamethasone sodium phosphate (DSP) is 0.5% of the price of Ozurdex. In the study by Fonseca et al., the effect of treating DME in patients with pseudophakia with the administration of DSP (4 mg/mL) at doses of 0.04 mg (0.01 mL), 0.12 mg (0.03 mL), and 0.20 mg (0.05 mL) was determined. A significant reduction in CMT was observed on the third postinjection day, with the therapeutic effect gradually diminishing over the first month. No significant differences in IOP measurements were observed^(^4^)^.

Through this letter we wish to bring to your attention a study we conducted to evaluate the response of patients with DME in the first 28 days following a single intravitreal injection of 0.08 mg (0.04 mL) of DSP (2 mg/mL). This Phase II clinical trial was approved by the Ethics Committee of the Hospital das Clínicas, Federal University of Pernambuco (No: 66509622.7.0000.8807) and registered in the Brazilian Clinical Trials Registry (No: RBR-59phz5c). The following were the inclusion criteria: adults with a diagnosis of diabetes mellitus for at least 5 years; presence of DME, which was defined as CMT >300 µm on optical coherence tomography that was caused by intraretinal or subretinal fluid accumulation; corrected distance visual acuity (CDVA) of 1.3 to 0.2 LogMAR; and a history of cataract surgery with intraocular lens implantation. After application of a topical anesthetic agent (proparacaine eye drops), the intravitreal injection was administered via the superior temporal quadrant of the pars plana, 3.5 mm from the limbus. The primary outcome was the reduction in CMT by the third postinjection day (D3). Secondary outcomes included the reduction in CMT on the seventh (D7) and twenty-eighth (D28) postinjection days, the change in CDVA on D3, D7, and D28, and the change in IOP on D3, D7, and D28.

**Figure 1:**
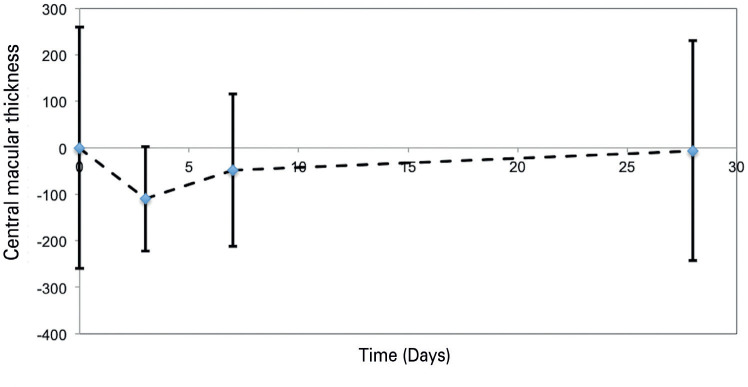
Central macular thickness after the administration of an intravitreal injection of dexamethasone sodium phosphate for the treatment of diabetic macular edema.

Our sample consisted of 12 volunteers (eight women) with a mean ± standard deviation age of 60 ± 6 years, mean diabetes diagnosis duration of 15 ± 6 years, and mean glycated hemoglobin level of 7.98 ± 1.19%. The central tendency data for CMT, CDVA, and IOP on D0, D3, D7, and D28 are presented in Table 1 and Figure 1. Nine of the 12 patients demonstrated a >10% reduction in CMT by D3. On an average, the CDVA improved by approximately one line of vision. No patient presented with an IOP >21 mmHg or required ocular hypotensive medications. No significant ocular or systemic adverse events were reported.

**Table 1:** Central macular thickness, corrected distance visual acuity, and intraocular pressure after intravitreal administration of dexamethasone sodium phosphate for the treatment of diabetic macular edema

	D0	D3	D7	D28	p1	p2	p3
	Median (IQR)	Median (IQR)	Median (IQR)	Median (IQR)			
CMT (µm)	435 (260)	325 (112)	387 (164)	429 (237)	.002	.002	.014
CDVA (LogMAR)	0.48 (0.45)	0.35 (0.45)	0.35 (0.50)	0.40 (0.28)	.107	.011	.011
IOP (mmHg)	15 (3)	17 (5)	16 (4)	17 (4)	.064	.621	.372

D0= preoperative data; D3= 3 days postinjection; D7= 7 days postinjection; D28= 28 days post-injection; IQR= interquartile range; CMT= central macular thickness; CDVA= corrected distance visual acuity; IOP= intraocular pressure; p1= Wilcoxon signed ranks test (D3-D0); p2= Wilcoxon signed ranks test (D7-D0); p3= Wilcoxon signed ranks test (D28-D0).

Thickness has been reported as median (interquartile range)

Our study’s results indicate that an intravitreal injection of 0.08 mg (0.04 mL) of DSP (2 mg/mL) in patients with DME reduces the CMT in the first 3-7 days after the injection. Additionally, the injection may facilitate some CDVA improvement without a significant increase in IOP.

Although this study was limited by its small sample size and the exclusion of phakic patients and patients with glaucoma, the results are promising. It demonstrated that DSP may be a cost-effective alternative or adjunct to current DME treatments in the following ways: to reduce macular thickness before macular laser photocoagulation, decrease the inflammatory response causing macular thickening after retinal laser photocoagulation, reduce macular edema if injected 3 days before the surgical removal of epiretinal membranes, and minimize the initial postoperative inflammatory response when injected during intraocular eye surgeries. Furthermore, DSP can be used as an adjuvant to anti-VEGF in the treatment of DME and a predictor of response to intravitreal steroid release system implants (Ozurdex).

This pilot study demonstrates the potential of DSP as a safe and effective treatment for DME. However, larger randomized controlled trials are required to confirm these findings.
